# Supplementary material for: TBHubbard: tight-binding and extended Hubbard model dataset for metal-organic frameworks
Source: Sci Data. 2025 Nov 12;12:1776. doi: 10.1038/s41597-025-06054-w (PMC12612188; doi:10.1038/s41597-025-06054-w)
Supplement: Supplementary file 1 — Supporting Information [file 41597_2025_6054_MOESM1_ESM.pdf]

Supporting Information

The distribution of the CPU run time for the TB and EH subsets, considering the executing of `hp.x` and `pw.x`, is plotted Fig. S1(a) and Fig. S1(b), respectively. The average runtime is represented as a dashed vertical line. Note the different orders of magnitude for the executions, where `hp.x` is extremely more costly than the `pw.x`.

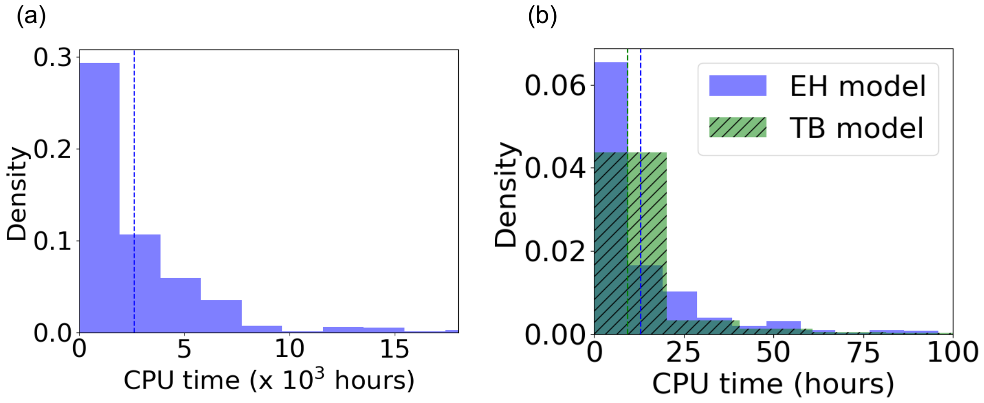

Fig. S1 Normalized histograms representing the CPU time in hours of the (a) Hubbard parameter computation using the executable `hp.x` from QE and (b) ground state calculation using the executable `pw.x` from Quantum Espresso (QE). Both subsets are accounted here, with 428 and 10345 calculations for the Extended Hubbard (EH), in blue, and Tight-binding (TB) subsets, in green hatched, respectively.

The energy difference between the DFT and DFT+U+V band gaps are plotted as a function of U and V in Fig. S2, for d-p and d-s perturbations. While a correlation might be drawn between the band gap energy difference and U, for V this behavior is not as clear.

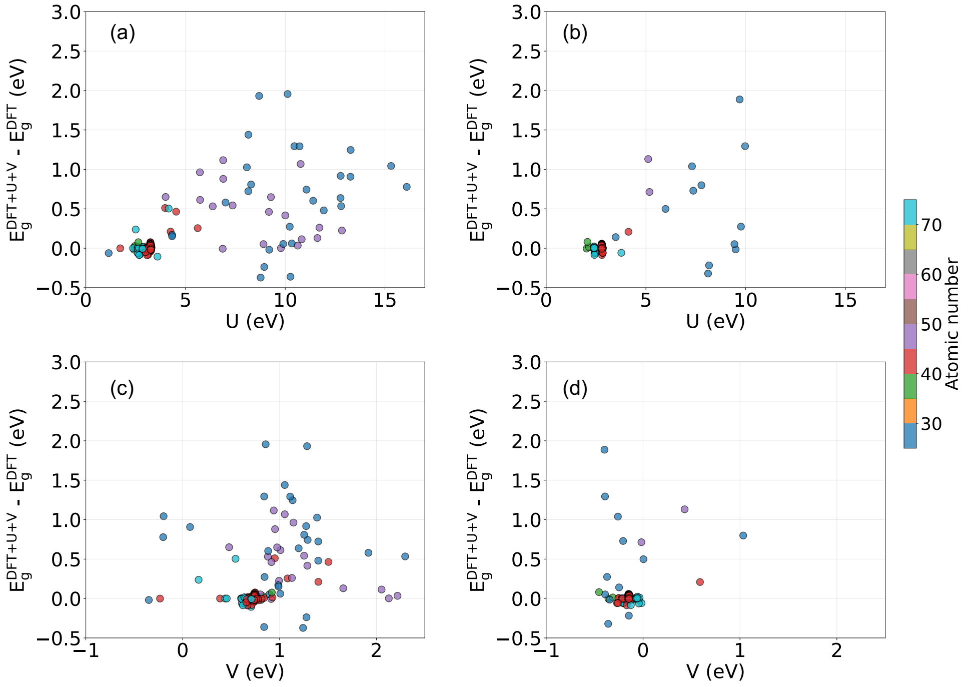

Fig. S2 Energy difference between band gaps computed with DFT (E_g^{DFT}) and DFT+U+V ($E_g^{DFT+U+V}$) as a function of U for dp (a) and ds perturbations (b) in the Extended Hubbard (EH) subset. The same is plotted as a function of V for dp (c) and ds perturbations (d), as well. The colormap represents the atomic number of the transition metal in each MOF.

To verify the accuracy of the tight-binding projection, in Fig. S3, we plot the DFT band structure obtained through QE for a particular MOF in our dataset, along with the eigenvalues of the TB Hamiltonian at Γ. From these results, we see that the electronic structure is well represented by the tight-binding projection for the k-point chosen here. Note that to reproduce the entire band structure with higher accuracy, a denser k-grid may be needed in the self-consistent step.

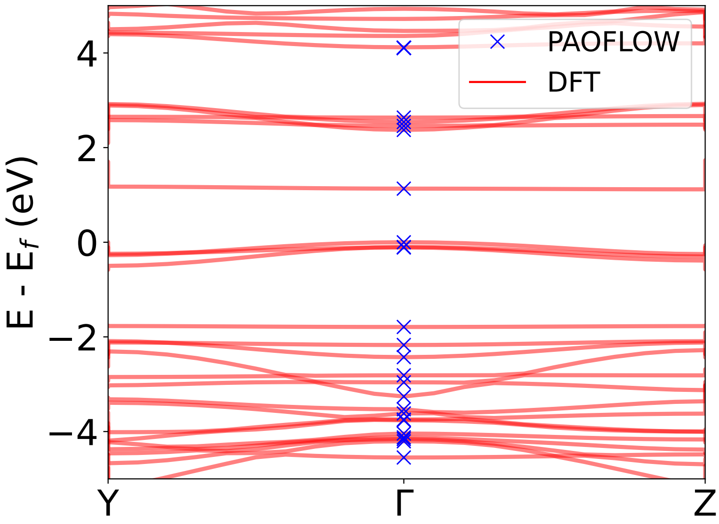

Fig. S3 Band structure of `qmof-3dfbcbd` computed through DFT with Quantum Espresso in solid red lines. The eigenvalues of the tight-binding matrix at Γ are plotted in blue.

Example of Structural Search Using TB and SOAP Embeddings. To demonstrate the utility of TB and SOAP embeddings, we begin by focusing on the TB embeddings of metal atoms across a specific MOF, as previously restricted for example purposes in the context of metal cluster design. Using the same train-test split from Fig. 5, from the main text, we select one metal from the test set, for which we have the ground truth. For this test, we treat the corresponding TB embedding vector as if it were predicted by a genetic algorithm. We then search for the closest TB embeddings within the training set and visually compare the resulting structures, as shown in Fig. S4. This search reveals a significant structural resemblance between the predicted and true structures.

Simultaneously, we predict the SOAP descriptors for this same metal atom. Since the corresponding SOAP descriptor is available in the ground truth of the test set, we compare the predicted SOAP descriptor to the true one, which differs only by a small error for SOAP-3Å and a marginally larger error for SOAP-5Å. We proceed to search for the closest vector in the SOAP space, which is nearer to the predicted descriptor rather than the true one. By doing so, we can identify similar Zr atoms across different MOFs, see Fig. S4. Furthermore, since SOAP descriptors are built with a reduced species set that maps all metals to a single category, we can perform a metal-agnostic query. This allows us to search for MOFs that exhibit the same local environment but with a different metal species, such as Hf, which we identify in the results, see Fig. S5.

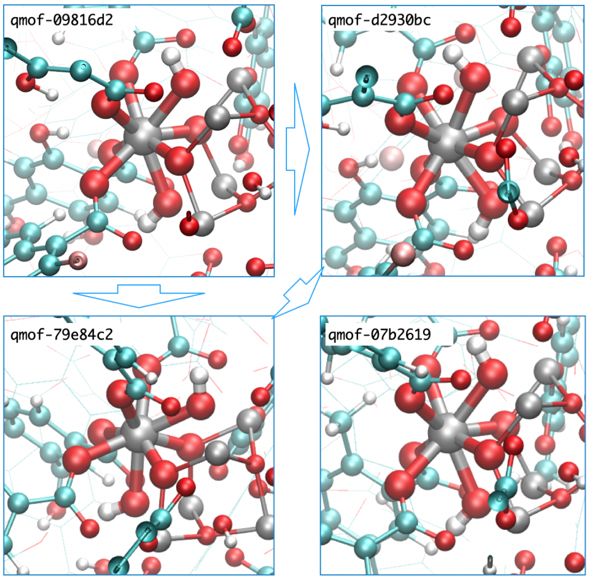

Fig. S4 Illustration of the initial structure from which the Tight-binding and SOAP embeddings search was conducted. The top left panel shows the selected metal atom within the MOF. Zr-atom in the `qmof-09816d2` (only present in the test set). The other three panels indicated by the arrow display the closest MOF structures based on the TB embeddings, highlighting the structural similarities to the initial configuration. Zr-atom in `qmof-d2930bc`, `qmof-79e84c2`, and `qmof-07b2619`.

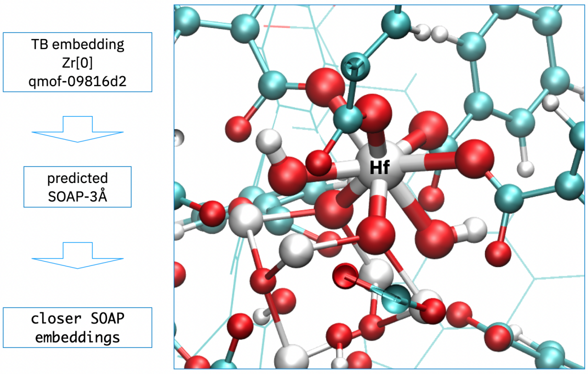

Fig. S5 A `RandomForestRegressor` predicts the SOAP-3Å embedding from the Tight-binding embeddings. The right panel presents one of the closest structures identified in the SOAP space with a different metal atom, demonstrating the metal-agnostic nature of SOAP descriptors. In this case, we identify an Hf atom in `qmof-75cdf73` as having a highly similar local environment.
